# Mechanisms of Psychological Distress following War in the Former Yugoslavia: The Role of Interpersonal Sensitivity

**DOI:** 10.1371/journal.pone.0090503

**Published:** 2014-03-21

**Authors:** Angela Nickerson, Stefan Priebe, Richard A. Bryant, Nexhmedin Morina

**Affiliations:** 1 School of Psychology, University of New South Wales, NSW, Sydney, Australia; 2 Unit for Social and Community Psychiatry, Barts' and the London School of Medicine and Dentistry, Queen Mary University of London, United Kingdom; 3 Department of Clinical Psychology, University of Amsterdam, The Netherlands; Harvard Medical School, United States of America

## Abstract

While high prevalence rates of psychological symptoms have been documented in civilian survivors of war, little is known about the mechanisms by which trauma exposure might lead to poor psychological outcomes in these populations. One potential mechanism that may underpin the association between war-related traumatic experiences and psychopathology is interpersonal sensitivity. In the current study, we applied structural equation modeling to investigate the impact of interpersonal sensitivity on posttraumatic stress disorder (PTSD) symptoms, depression symptoms, and anger responses following exposure to war trauma. 3313 survivors of the war in the former Yugoslavia were identified and selected using a multistage, probabilistic sampling frame and random walk technique. Participants were interviewed regarding trauma exposure, interpersonal sensitivity, and PTSD symptoms, depression symptoms, and anger responses. Structural equation modeling analyses revealed that the relationship between trauma and PTSD symptoms and depression symptoms was partly statistically mediated by interpersonal sensitivity. Further, findings indicated that the relationship between trauma and anger responses was fully statistically mediated by interpersonal sensitivity. These results suggest that interpersonal sensitivity may function as a key mechanism that contributes to psychopathology following trauma.

## Introduction

Research has consistently documented elevated rates of psychological disorders in civilian survivors of war trauma [1]–[3]. A meta-analysis of 181 studies conducted across the globe estimated that approximately 30% of conflict-affected civilians and refugees meet criteria for posttraumatic stress disorder (PTSD) and/or depression [2]. While exposure to war trauma is associated with elevated rates of psychopathology, individuals meeting criteria for disorder in conflict-affected groups are usually in the minority. This highlights the considerable variation in how well people adapt psychologically following exposure to war-related trauma. Research has identified contextual factors, such as type and/or dosage of trauma exposure and post-trauma stressors, which impact on the variable psychological outcomes in conflict-affected groups [4]–[6]. In contrast, relatively little research attention has been paid to the psychological mechanisms that contribute to the development and maintenance of psychological symptoms following trauma exposure in war-affected populations.

Traumatic events that occur in the context of war and persecution are often repeated and human-instigated, such as witnessing the violent death of loved ones, being beaten or seriously injured by another person, or being tortured. In addition to poor mental health outcomes, exposure to interpersonal trauma has been linked to negative social consequences including impaired capacity to relate to others and decreased interpersonal trust [7], [8]. One specific mechanism by which human-instigated trauma has been demonstrated to influence mental health and social functioning is interpersonal sensitivity, defined as “undue and excessive awareness of, and sensitivity to, the behaviour and feelings of others” [9]. Studies suggest that interpersonal sensitivity is associated with exposure to various types of trauma, including childhood abuse [10], dating violence [11], and war trauma [4]. Individuals who have experienced multiple traumatic events evidence higher levels of interpersonal sensitivity than those who have experienced a single traumatic event [12]. Further, interpersonal sensitivity has been linked to negative mental health outcomes including PTSD, depression, and anxiety [4], [13]–[16].

Researchers and clinicians have noted that difficulty trusting others and increased perceptions of hostility are common phenomena in refugee and post-conflict populations [17]–[20]. Indeed, these responses may be adaptive in the context of war and persecution, where heightened awareness of the intentions of others is likely to facilitate the identification (and adaptive avoidance) of interpersonal threat. In conflict-affected settings, where misplaced trust may have catastrophic consequences, sensitivity to potential interpersonal threat may remain high, even when there is no longer imminent danger. This heightened sensitivity may be unnecessary or even contribute to psychological distress, as evidenced by research findings linking interpersonal sensitivity and psychological symptoms [4], [15], [16]. Further, interpersonal sensitivity is likely to impact on interpersonal behaviors and social functioning. One plausible consequence of interpersonal sensitivity is heightened anger reactions, as rumination on trauma and injustice may precipitate anger reactions in response to perceived threat. This may be especially salient in conflict-affected settings where preoccupation with past injustices and the desire for revenge is common [21], [22]. Accordingly, emerging research suggests that anger reactions are highly prevalent amongst war survivors and refugees [23]–[25], and are associated with exposure to human rights violations and socio-economic factors [24], [25]. The enormous social and healthcare cost of anger and violence, and the recognized relationship between anger, trauma and PTSD [26]–[28] necessitates further research on anger responses and their underlying mechanisms.

Based on research documenting the deleterious impact of war trauma on mental health [1]–[3] and that linking interpersonal sensitivity with both trauma exposure and psychological symptoms [4], [15], [16], the aim of the current study was to investigate the association between trauma exposure, interpersonal sensitivity and psychological outcomes. Participants were trauma-exposed survivors of wars in the former Yugoslavia, drawn from five countries in the Balkans region, and interviewed an average of eight years since the end of the war. It was hypothesized that interpersonal sensitivity would partially statistically mediate the association between trauma exposure and PTSD symptoms, depression symptoms, and anger reactions in civilian survivors of wars in the former Yugoslavia.

## Methods

### Procedure

Full details about the rationale of the study and its methods have been published elsewhere [3]. This study was conducted in 2006 and 2007. Face-to-face interviews were conducted by interviewers trained in the assessment measures. A multistage, probabilistic sampling frame and random walk technique was used to identify and select participants. Administrative regions that had been directly exposed to war for at least seven days were first selected. Following this, 20% of these regions were randomly selected (with a minimum of two administrative regions per country). Overall, 15 regions were selected across the five participating countries. Three localities with a minimum population of 3,000 per locality were randomly selected in each of these regions, resulting in a total selection of 49 locations across all countries. To avoid oversampling from the most populous localities, it was ensured that a maximum of 25% of the study sample in each country was recruited in a single locality.

Streets were randomly identified in each locality, and every fourth household was selected up until a maximum of 15 interviews per street were completed. Households in the same building were randomly chosen and it was ensured that no more than six participants per building were interviewed. The eligible adult member of the household whose birthday was closest to the date of the interview was interviewed. Inclusion criteria were: born within the former Yugoslavia, aged 18 to 65 years, experienced a minimum of one war-related potentially traumatic event, last war-related event experienced at ages 16 or older, no severe learning difficulty, and no mental impairment relating to brain injury or other organic cause. Potential participants were initially screened using a list of 20 war-related stressful events.

### Participants

In total, 3313 war survivors residing in the following countries were interviewed, 640 (19.32%) in Bosnia and Herzegovina, 727 (21.94%) in Croatia, 648 (19.56%) in Kosovo, 661 (19.98%) in Macedonia, and 637 (19.23%) in Serbia. Participants had a mean age of 42.52 (SD = 12.01), and consisted of 1529 males (46.17%) and 1783 females (53.83%). Participants had completed a mean of 10.91 (SD = 3.47) years of education. Participants had been exposed to war between five and 15 years previously. During the war, 1820 participants had remained at home (55.05%), 887 participants had been displaced within the former Yugoslavia (26.83%), and 599 were refugees (18.12%). Furthermore, 578 participants had been actively involved in combat (17.4%). Participants' marital status was as follows: married N = 2296 (66.39%), single N = 606 (18.31%), divorced N = 176, (5.32%), widowed N = 202 (6.10%), living with partner N = 29, (0.88%). Participants' employment status was as follows: in paid employment N = 1188 (77.50%), student N = 141 (4.26%), retired N = 439 (13.27%), unemployed N = 1539 (46.51%).

### Ethics statement

We obtained written informed consent from participants before the interview. The study was approved by the Royal Free Medical School Research Ethics Committee (REC reference number 04/QO501/118).

### Measures

An adapted version of the Life Stressor Checklist, Revised was used to assess trauma exposure [29]. This list consisted of 24 potentially traumatic experiences, and participants responded regarding whether they had experienced each event before, during, and after the war. In the current study, we used a total score of the number of types of traumatic events participants had experienced during the war.

The Brief Symptom Inventory [30] is a 53-item scale measuring various domains of psychological distress experienced in the previous week. Items are scored on a five-point response scale ranging from 0 (not at all) to 4 (extremely). The four-item interpersonal sensitivity subscale was used to index levels of interpersonal sensitivity (e.g., *“Feeling that people are unfriendly or dislike you”*); the six-item depression subscale was used to measure depression symptoms (e.g., *“Feeling no interest in things”*), and the four-item hostility subscale was used to index anger reactions (e.g., *“Having urges to beat, injure or harm someone”*). The authors reported good test-retest reliability for the general severity index (.90) and the nine BSI subscales (.68–.91). In the current study, the BSI demonstrated adequate internal consistency values for each of the three subscales ranging (depression *α* = 0.83, hostility *α* = 0.77, interpersonal sensitivity *α* = 0.80).

The Impact of Events Scale – Revised [31] was used to assess symptoms of PTSD. These symptoms were anchored to war-related traumatic experiences.This 22-item scale indexes severity of PTSD symptoms, with responses ranging from 0 (not at all) to 4 (extremely). Total symptom scores for each of the DSM-IV PTSD symptom clusters (re-experiencing, avoidance, hyperarousal) were calculated by summing the relevant items. The authors reported high test–retest reliabilities and internal consistencies of the three subscales, with alpha coefficients ranging from 0.79 to 0.92, In the current study, the IES-R demonstrated high internal consistency values for the total scale as well as the three subscales ranging from *α* = 0.92 to *α* = 0.95.

The Mini-International Neuropsychiatric Interview (MINI) [32] was used to assess diagnostic caseness for PTSD and depression in this study.

### Data analysis

Pearson bivariate correlations were first calculated to investigate inter-variable correlations. We then applied structural equation modeling using Mplus Version 7 [33] to examine hypothesized models of the relationships between variables. Mplus implements a robust full information maximum likelihood estimation procedure to account for missing data. We first evaluated the measurement model to assess the extent to which latent variables were represented by indicator variables. We used the four BSI-Interpersonal Sensitivity Items as indicators for the interpersonal sensitivity latent variable. We used the sum total of each of the DSM-IV PTSD symptom clusters (re-experiencing, avoidance, and hyperarousal symptoms) to represent PTSD symptoms, and the six BSI-Depression items as indicators for the depression symptoms latent variable. Finally, we used the four BSI-Hostility items as indicators for the anger latent variable. Total number of types of trauma to which the individual was exposed during the war constituted an observed variable.

**Figure 1:**
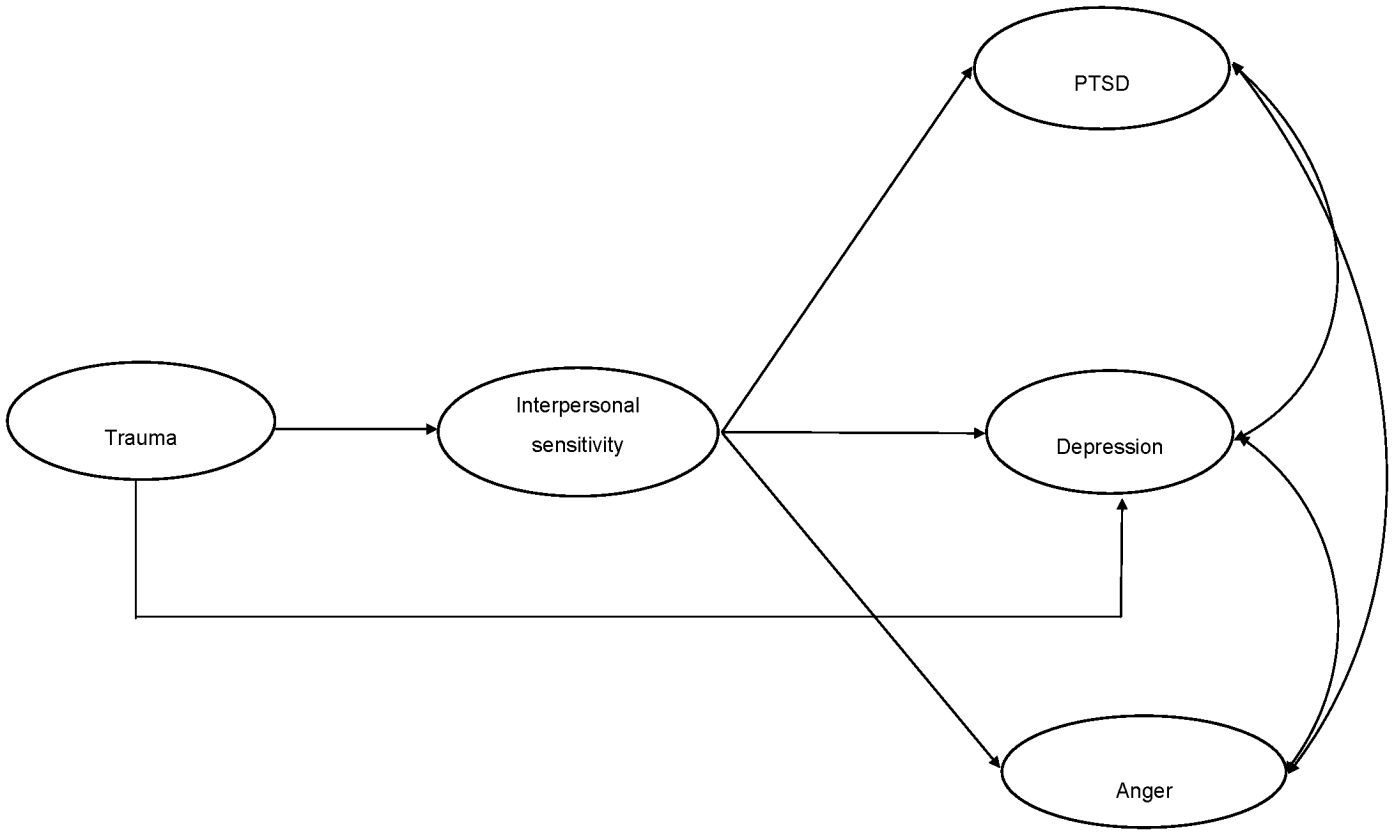
Full mediation model of association between trauma, interpersonal sensitivity, PTSD, depression, and anger.

**Figure 2:**
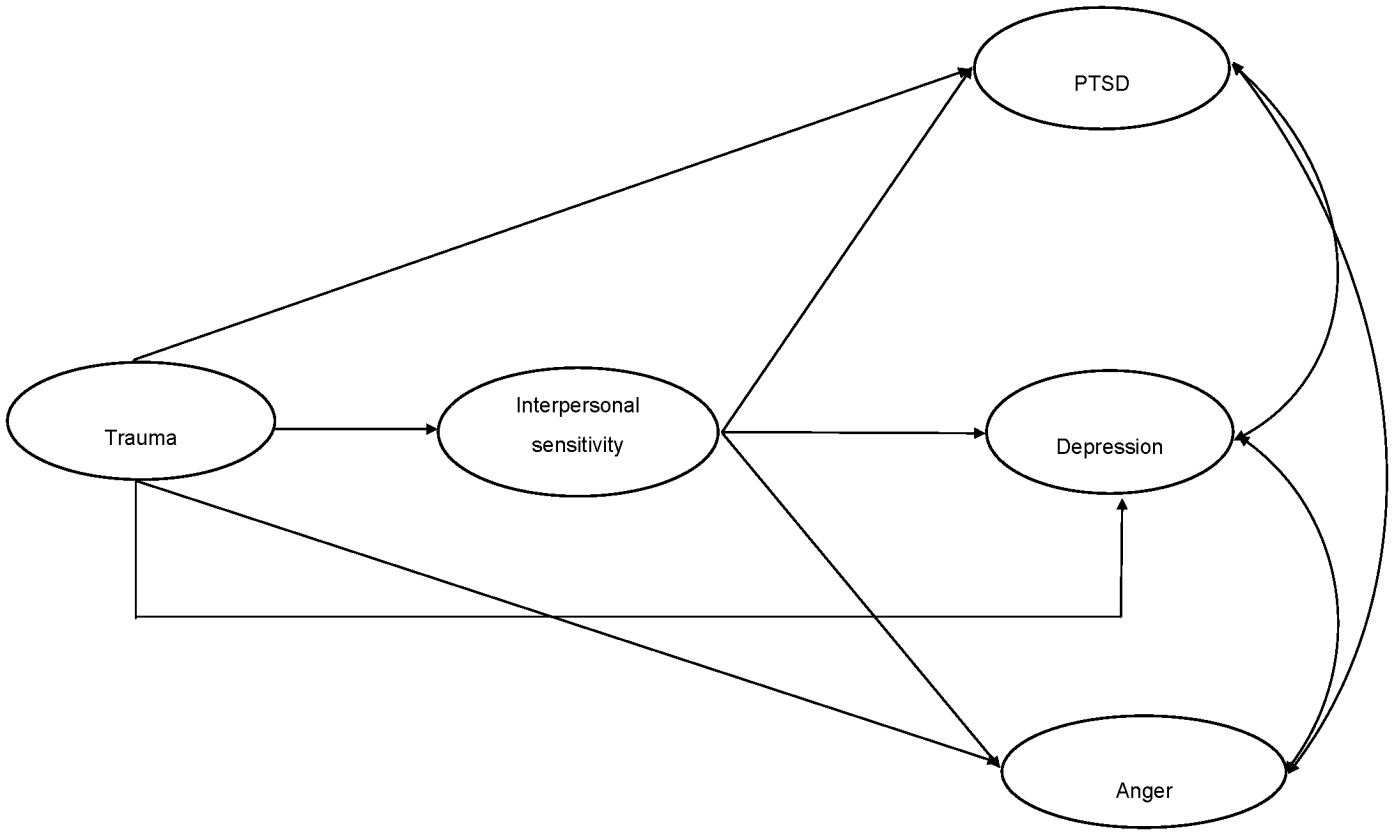
Partial mediation model A of association between trauma, interpersonal sensitivity, PTSD, depression, and anger.

After determining the measurement model evidenced adequate fit, we tested three structural models to examine the relationship between trauma exposure, interpersonal sensitivity, and psychological outcomes (PTSD symptoms, depression symptoms, and anger reactions). In Model 1 (presented in Figure 1) the relationship between trauma exposure and psychological outcomes (PTSD symptoms, depression symptoms and anger reactions) was statistically mediated in full by interpersonal sensitivity. In Model 2 (presented in Figure 2), the relationship between trauma exposure and psychological outcomes was statistically mediated in part by interpersonal sensitivity. We also tested models in which the relationship between trauma exposure and interpersonal sensitivity was statistically mediated by psychological outcomes (PTSD symptoms, depression symptoms, and anger responses) both in full (CFI = 0.87, TLI = 0.84, RMSEA = 0.11, SRMR = 0.23) and in part (CFI = 0.86, TLI = 0.84, RMSEA = 0.11, SRMR = 0.23), however these models evidenced poor fit to the data, and thus we did not pursue this line of enquiry. SEM models were also tested across gender. As very similar patterns of results emerged for males and females, we focused on the complete sample for the current analyses.

We evaluated the goodness-of-fit of the model using the following indices: (1) Root Mean Square Error of Approximation (RMSEA) <0.06); (b) Standardized Root Mean Square Residual (SRMR) <0.08; and (c) Comparative Fit Index (CFI) and Tucker-Lewis Index (TLI) values approaching .95 or greater [34]. As the chi-square statistic is influenced by sample size, such that models are more likely to be erroneously rejected as a function of large sample size [35], the chi-square statistic was only used to index relative fit of various models (rather than absolute fit) in this study.

## Results

Frequencies of exposure to specific types of traumatic events within the sample are presented in Table 1. Participants reported an average of 8.6 (SD = 3.4) years since the most traumatic war-related event. 19.9% (N = 657) of the sample met diagnostic criteria for a Major Depressive Episode, and 20.1% (N = 665) met diagnostic criteria for PTSD. We examined correlations between total scores on each variable as a preliminary investigation of relationships between variables in this study. All measured variables were significantly correlated with each other (see Table 2).

**Table 1:** Trauma exposure reported by 3313 survivors of war in the former Yugoslavia.

Trauma type	*n*	%
Serious accident	200	6.04%
Natural disaster	36	1.09%
Assault by family member	79	2.39%
Assault by stranger	241	7.28%
Sexual assault by family member	4	0.12%
Sexual assault by stranger	8	0.24%
Imprisonment	139	4.20%
Life threatening illness	105	3.17%
Sudden death of a dear person	290	8.75%
Lack of food and water	1222	36.90%
Ill without medical care	274	8.27%
Lack of shelter	1694	51.40%
Expelled from home	1267	38.25%
Combat situation	544	16.44%
Shelling/bombardment	2798	84.61%
Siege	1329	40.14%
Serious injury	226	6.82%
Witnessed an assault, murder or death	791	23.98%
Learned about murder of a dear person	1187	35.95%
Disappearance or kidnapping of a family member	192	5.80%
Torture	287	8.66%
Lost	287	8.67%
Kidnapped	100	3.02%
Mine explosion	233	7.30%

**Table 2:** Descriptive statistics and correlations of study measures.

		Mean	SD	1	2	3	4	5
1	Trauma exposure	4.05	2.71	-				
2	Interpersonal sensitivity	2.68	3.22	0.19*	-			
3	PTSD	24.05	23.17	0.26*	0.53*	-		
4	Depression	4.63	5.13	0.28*	0.75*	0.64*	-	
5	Anger	2.51	3.00	0.16*	0.62*	0.53*	0.64*	1

*p<.001.

### Measurement model

The initial measurement model evidenced reasonable fit (CFI = 0.94, TLI = 0.92, RMSEA = 0.07, SRMR = 0.05), however modification indices suggested that the residual variances of certain indicator variables be correlated. In the interpersonal sensitivity latent variable, this included *feelings easily hurt* and *feeling that others dislike you;* in the depression latent variable, this included *feeling lonely* and *feeling blue*; and in the anger latent variable, this included *urges to beat, injure or harm others* and *urges to break or smash things*. Correlating the residual variance of indicator variables suggests that these pairs of variables are related due to variables not included in the model. This was theoretically consistent with the specific pairs of variables to be correlated, thus we evaluated a second measurement model in which the residual variances of these indicator variables were correlated. This resulted in improved model fit (*χ^2^Δ* (7) = 890.48, *p*<.001 CFI = 0.96, TLI = 0.95, RMSEA = 0.06, SRMR = 0.03), thus this model was retained for subsequent analyses. Means, standard deviations, and factor loadings for study variables are presented in Table 3.

**Table 3:** Standardized factor loadings for interpersonal sensitivity, PTSD symptoms, depression symptoms, and anger symptoms from measurement model.

Variable	Factor loading
Interpersonal sensitivity	
Your feelings being easily hurt	0.69
Feeling that people are unfriendly or dislike you	0.66
Feeling inferior to others	0.74
Feeling very self-conscious with others	0.68
PTSD symptoms	
Re-experiencing	0.97
Avoidance	0.87
Hyperarousal	0.95
Depression symptoms	
Thoughts of ending your life	0.46
Feeling lonely	0.77
Feeling blue	0.73
Feeling no interest in things	0.78
Feeling hopeless about the future	0.74
Feelings of worthlessness	0.76
Anger symptoms	
Feeling easily annoyed or irritated	0.70
Having urges to beat, injure, or harm someone	0.62
Having urges to break or smash things	0.65
Getting into frequent arguments	0.70

### Comparative testing of structural models

**Figure 3:**
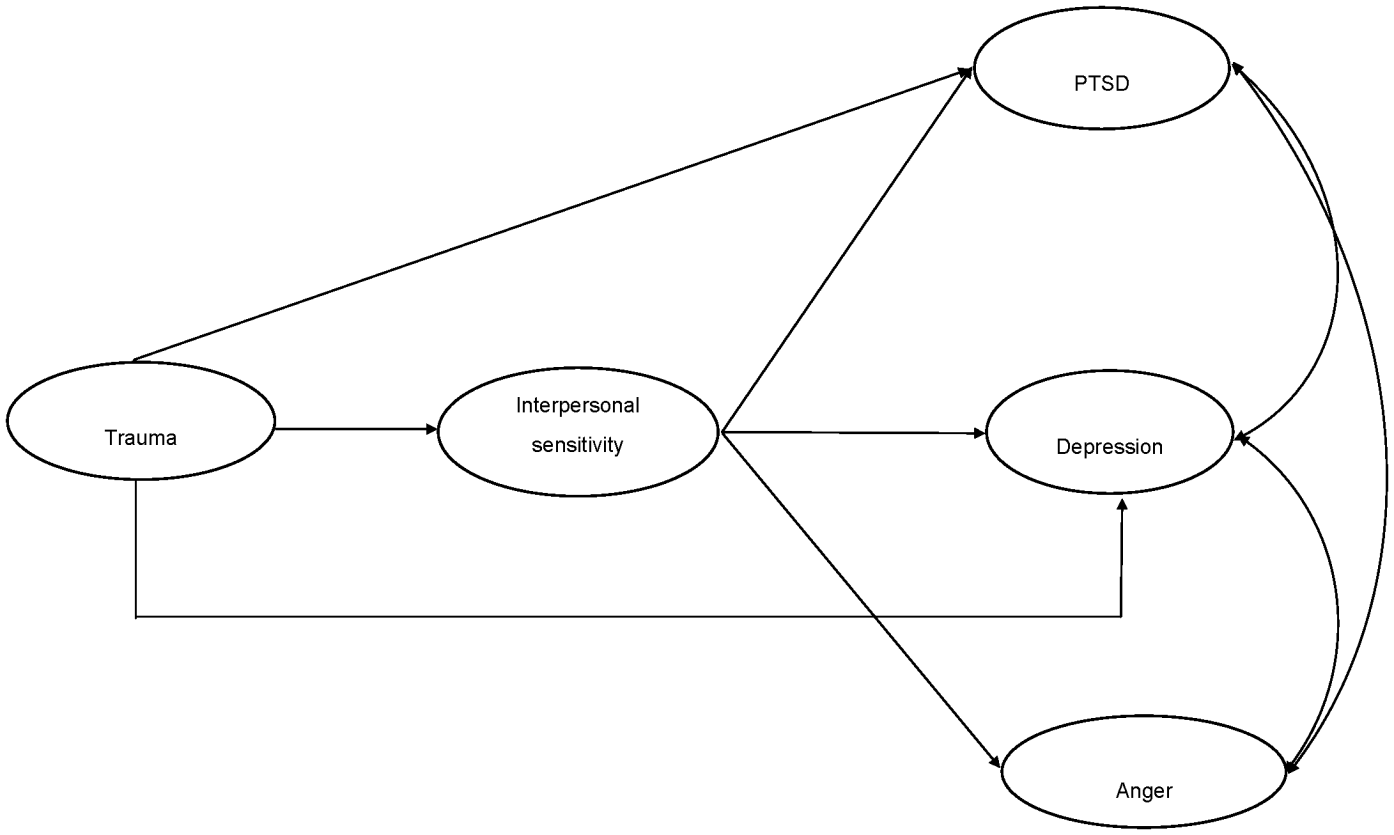
Partial mediation model B of association between trauma, interpersonal sensitivity, PTSD, depression, and anger.

**Figure 4:**
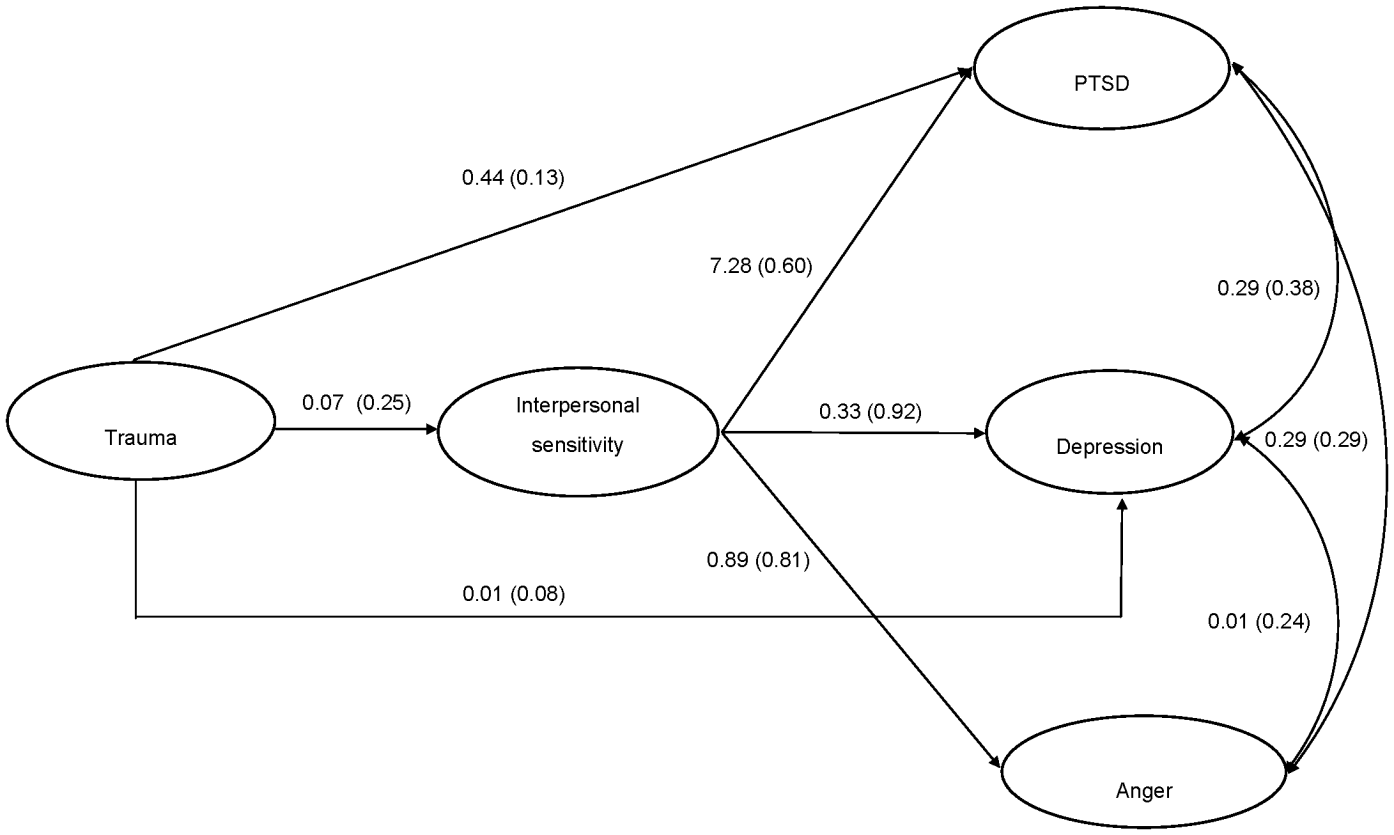
Partial mediation model B of association between trauma, interpersonal sensitivity, PTSD, depression, and anger. Unstandardized coefficients followed by standardized coefficients in parentheses are presented.

Both of the initial structural models evidenced good fit: Model 1 (Figure 1) *χ^2^* (126) = 1629.57, *p*<.001, CFI = 0.96, TLI = 0.95, RMSEA = 0.06, SRMR = 0.04; Model 2 (Figure 2) *χ^2^* (123) = 1543.74, *p*<.001, CFI = 0.96, TLI = 0.95, RMSEA = 0.06, SRMR = 0.03. Comparative model testing revealed that Model 2 fit the data significantly better than Model 1 (*χ^2^Δ* (4) = 95.31, *p*<.001). This indicates that interpersonal sensitivity did not fully statistically mediate the association between trauma exposure and psychological outcomes. In Model 2, all paths were significant, with the exception of the pathway between trauma exposure and anger reactions. Upon removal of this path, the model (Model 3, Figure 3) continued fit the data well: *χ^2^* (124) = 1544.118, *p*<.001, CFI = 0.96, TLI = 0.95, RMSEA = 0.06, SRMR = 0.03). We then compared Model 2 and Model 3 to determine whether the removal of the structural path between trauma exposure and anger significantly harmed overall model fit. Comparison of Model 2 and Model 3 indicated that there was no significant difference in model fit (*χ^2^Δ* (1) = 0.38, *ns*). Model 3 was selected as the best model as it represented the most parsimonious model with the best fit. Final unstandardized and standardized path estimates for Model 3 are presented in Figure 4.

Next we tested the significance of indirect effects in Model 3. Standardized total, direct, and indirect effects, in addition to 95% confidence intervals, are displayed in Table 4. Analyses indicated that there was a significant indirect effect from trauma exposure to PTSD symptoms via interpersonal sensitivity (Estimate = 0.15, Standard Error (SE) = 0.01, *p*<.001, 95% Confidence Interval (CI) = 0.13 to 0.17). Further, there was a significant indirect effect from trauma exposure to depression symptoms via interpersonal sensitivity (Est = 0.23, SE = 0.02, *p*<.001, 95% CI = 0.20 to 0.26). Finally, there was a significant indirect effect from trauma exposure to anger reactions (Est = 0.20, SE = 0.02, *p*<.001, 95% CI = 0.18 to 0.23).

**Table 4:** Standardized total, direct, and indirect effects from trauma to PTSD, depression and anger.

	*Est*	*SE*	*p*	95%CI
Trauma exposure to PTSD				
Total	0.28	0.02	<.001	[0.25, 0.30]
Direct	0.13	0.02	<.001	[0.10, 0.15]
Indirect	0.15	0.01	<.001	[0.13, 0.17]
Trauma exposure to depression				
Total	0.30	0.02	<.001	[0.28, 0.33]
Direct	0.08	0.01	<.001	[0.05, 0.08]
Indirect	0.23	0.02	<.001	[0.20, 0.26]
Trauma exposure to anger				
Total	0.20	0.02	<.001	[0.18, 0.23]
Direct	-	-		
Indirect	0.20	0.02	<.001	[0.18, 0.23]

## Discussion

This study investigated the relationship between trauma exposure, interpersonal sensitivity, and psychological outcomes in a sample of over 3,000 war survivors. The key finding from this study was that interpersonal sensitivity fully statistically mediates the relationship between war trauma exposure and anger reactions, and partially statistically mediates the association between trauma exposure and symptoms of PTSD and depression. These results extend upon research conducted with non-war survivors indicating that trauma exposure (especially interpersonal trauma) is associated with higher levels of interpersonal sensitivity [11], [12]. This is consistent with research suggesting that interpersonal sensitivity is associated with PTSD symptoms [13], [36], as well as other types of psychopathology [14]–[16]. For example, a longitudinal study conducted with Vietnamese refugees indicated that those with PTSD had significantly higher levels of interpersonal sensitivity than those without PTSD both upon arrival to the resettlement country and at a three year follow-up assessment [4]. It is notable that, in the current study, the impact of trauma exposure on psychological outcomes via interpersonal sensitivity was equal to (in the case of PTSD symptoms) or stronger than (in the case of depression symptoms and anger reactions) the direct effect of trauma exposure. This highlights the importance of the association between interpersonal sensitivity and mental health outcomes in survivors of war trauma.

The relationship between war-trauma and PTSD and depression is well-documented, with findings from research studies attesting to the dose-response relationship between trauma and psychological outcomes [37], [38]. Results from this study provide evidence for a potential mechanism that may underlie this relationship, suggesting that exposure to traumatic events may contribute to heightened interpersonal sensitivity, which is associated with poor psychological outcomes. While the impact of trauma exposure on psychological processes has attracted considerable research attention in western countries, relatively less research has been conducted considering psychological mechanisms in post-conflict settings. This research provides evidence that the psychological impact of trauma experienced in the context of war may extend beyond the event itself, to encompass post-trauma cognitive responses that shape future interactions and experiences; and ultimately contribute to mental health-related functioning.

Participants in the current study reported high levels of exposure to interpersonal trauma in the war in the former Yugoslavia, including such events as being assaulted, witnessing the assault or injury of others, and witnessing or learning of the murder of loved ones. It may be expected that exposure to repeated evidence of human malevolence in the form of interpersonal trauma may negatively impact on the individual's capacity to trust others and, ultimately, on their social functioning. This is consistent with evidence that traumatic events occurring in the context of war and persecution may negatively impact beliefs about the benevolence of humankind [39], and that difficulty trusting others is common in survivors of interpersonal trauma and human rights violations [20], [40], [41].

Interpersonal sensitivity may represent one mechanism by which distrust and psychological distress are linked. Accordingly, research indicates that interpersonal sensitivity is negatively correlated with beliefs about the benevolence of the world and other people [42]. It may be that exposure to repeated evidence of the capacity of humans to engage in violent and unpredictable behavior disrupts previously-held adaptive social beliefs and expectations [43]. This is in accordance with the assertion that exposure to interpersonal trauma challenges the perception that human behaviour is guided by social rules [44]. This may result in the individual becoming hypervigilant to the intentions and feelings of others, in attempt to protect himself or herself from further harm [12], [45]. Even after the trauma has ceased, this response may persist, as is consistent with the observation that interpersonal mistrust is a common long-term posttraumatic outcome [17], [20], [40], [46].

The finding that interpersonal sensitivity fully statistically mediated the relationship between trauma exposure and anger reactions in the current study has important implications for traumatic stress models. Recent research indicates that anger reactions, including intermittent explosive disorder, are prevalent in war-affected populations [24], [47], [48]. The link between anger and aggression and interpersonal sensitivity following trauma exposure indicates that interpersonal perceptions and expectations are likely to influence subsequent interpersonal interactions. Cycles of violence models note that individuals who have been exposed to violence are more likely to enact violence themselves [49], [50]. Interpersonal sensitivity may be an important mechanism underpinning the perpetuation of violence in traumatized individuals and societies. It is possible that sensitivity towards the behaviors of others may contribute to preoccupation with past injustice by increasing the likelihood that others' actions and intentions are perceived as threatening or negative, which may lead to aggressive retaliatory responses. Further research should investigate the role of interpersonal sensitivity in contributing to the perpetuation of cycles of violence in conflict-affected settings.

Strengths of the study include the multi-stage probabilistic sampling frame and random walk approach as well as the consistent methodology across several countries, including civilians and people with combat experience. Yet, there are several limitations associated with the current study. First, the cross-sectional design of this study precludes inferences about causality; for example, it may be that interpersonal sensitivity is a stable trait that precedes trauma exposure and influences mental health outcomes or it may be a function of trauma exposure and associated distress. Longitudinal research should be conducted to disentangle the temporal sequencing of trauma exposure, interpersonal sensitivity, psychopathology and impairments in social functioning. Second, past experiences were retrospective and may have been influenced by recall bias [51]. Third, the number of items measuring interpersonal sensitivity and anger was limited; a more comprehensive examination of these constructs, would facilitate the elucidation of more subtle interrelationships between these constructs. Fourth, we did not examine physical aggression or social functioning in this study, both of which are important constructs associated with anger and interpersonal sensitivity. Further research should investigate the association between interpersonal sensitivity, anger, and physical or verbal aggression to map the social consequences of the psychological effects of war trauma. In addition, further research should examine potential moderating variables of relationships between trauma exposure, interpersonal sensitivity, and psychological outcomes (e.g., age, socio-economic status, education).

Findings from the current study underscore the importance of investigating the influence of psychological processes on adaptation in conflict-affected populations. Future research should extend beyond documenting prevalence rates to examine psychological processes that may be implicated in adaptation following mass trauma. This will not only elucidate pathways to psychopathology and resilience, but also potentially inform the development of psychological interventions that directly target these processes.
